# Measurement of the Temperature Using the Tomographic Representation of Thermal States for Quadratic Hamiltonians

**DOI:** 10.3390/e23111445

**Published:** 2021-10-31

**Authors:** Julio A. López-Saldívar, Margarita A. Man’ko, Vladimir I. Man’ko

**Affiliations:** 1 Instituto de Ciencias Nucleares, Universidad Nacional Autónoma de México, Apdo. Postal 70-543, Ciudad de México 04510, Mexico; 2 Moscow Institute of Physics and Technology, Institutskii per. 9, Dolgoprudnyi, Moscow 141700, Russia; mankovi@lebedev.ru; 3 Russian Quantum Center, Skolkovo, Moscow 143025, Russia; 4 Lebedev Physical Institute, Leninskii Prospect 53, Moscow 119991, Russia; mankoma@lebedev.ru

**Keywords:** Wigner function, tomogram, Gaussian states, temperature, thermal states, coupled harmonic oscillators

## Abstract

The Wigner and tomographic representations of thermal Gibbs states for one- and two-mode quantum systems described by a quadratic Hamiltonian are obtained. This is done by using the covariance matrix of the mentioned states. The area of the Wigner function and the width of the tomogram of quantum systems are proposed to define a temperature scale for this type of states. This proposal is then confirmed for the general one-dimensional case and for a system of two coupled harmonic oscillators. The use of these properties as measures for the temperature of quantum systems is mentioned.

## 1. Introduction

The description of the state of a system is one of the most important problems of theoretical physics. For classical systems, states of a particle are described by its position *q* and momentum *p*. For a particle interacting with an environment, the states are described by a probability distribution f(p,q) in the phase space [1]. When quantum phenomena were discovered, a new notion of the particle state was introduced. Namely, the complex wave function $\psi \left(q\right)=\left|\psi \left(q\right)\right|e^{i\varphi (q)}$ was introduced for an isolated particle [2], and the density matrix $\rho (q,q^{\prime })$ (associated to a density operator $\hat{\rho }$) was introduced [3,4] to describe the particle states interacting with an environment. The evolution of states for isolated classical particles is described by its trajectory q(t) and p(t) in the phase space. For a classical particle interacting with the environment, the state evolution is associated with varying probability distribution f(q,p,t) in the phase space. It is important that, for the particle with Hamiltonian H(q,p) in the environment with temperature *T*, the thermal equilibrium state is associated with the probability distribution function $f\left(q,p,t\right)=\frac{e^{-H(q,p)/T}}{\int e^{-H(q,p)/T}\;dq\;dp}.$ In the case of a quantum particle, the state of a single particle evolving with time is given by the wave function ψ(q,t) satisfying the Schrödinger equation. For a quantum particle interacting with the environment, the state evolution is described by the density operator $\hat{\rho }\left(t\right)$ satisfying either the von Neumann equation [5] or the Gorini–Kosakowski–Sudarshan–Lindblad equation [6,7,8] for the density operator. In the case of thermal equilibrium, the state of the particle with Hamiltonian $\hat{H}\left(\hat{q},\hat{p}\right)$ is described by the density operator $\hat{\rho }\left(T\right)=\frac{e^{-\hat{H}\left(\hat{q},\hat{p}\right)/T}}{\mathrm{Tr}\;e^{-\hat{H}\left(\hat{q},\hat{p}\right)/T}}.$ We see that all discussed states of a quantum particle are described quite differently from the states of a classical particle. To make the description of quantum states similar to the description of classical states, different representations like the Wigner function W(q,p) [9], Husimi Q(q,p) function [10], and Glauber–Sudarshan *P* function [11,12] were introduced, but all these functions are not the probability distributions of the position *q* and momentum *p*, as this probability distribution does not exist in nature due to the Heisenberg uncertainty relation [13]. Nevertheless, it was recently found that there exists the representation of quantum states by means of the conditional probability distribution function W(X∣μ,ν) of one random variable *X* [14]. This representation also exists for classical particles and has the form of the Radon transform [15] of the probability density f(q,p), namely, W(X∣μ,ν)=∫f(q,p)δ(X−μq−νp)dqdp. This transform has the inverse, i.e., $f\left(q,p\right)=\frac{1}{4\pi^{2}}\int W\left(X|\mu ,\nu \right)\exp\left[-i\left(X-\mu q-\nu p\right)\right]\;dX\;d\mu \;d\nu .$ Thus, the states of both classical and quantum particles can be described by the probability distribution called the symplectic tomogram [14]; this tomogram is related to all the other quasidistributions for quantum particles. For example, the Wigner function known for a quantum state is related by the same Radon transform to the symplectic tomogram of the state with density operator $\hat{\rho }$, which determines the quantum particle tomogram, i.e., $W_{\rho }\left(X|\mu ,\nu \right)=\mathrm{Tr}\left(\hat{\rho }\cdot \delta \left(X\hat{1}-\mu \hat{q}-\nu \hat{p}\right)\right).$ On the other hand, it is worth studying thermal-equilibrium states and explicitly showing the tomograms associated with these states. For example, the states of particles with Hamiltonians, which are quadratic forms in the position $\hat{q}$ and momentum $\hat{p}$ operators, have attracted some attention in the literature [16].

The thermodynamic properties of quantum systems have been an important topic of research given the statistical implications concluded from them and the applications both theoretical and experimental. The main example of this is the Bose–Einstein condensate, which has provided an enormous amount of attention and applications for decades [17].

On the other hand, the research of quadratic systems has been important for the development of quantum mechanics as some of them have technological applications, such as the coherent and squeezed states. Furthermore, some important Hamiltonians are written in a quadratic form such as coupled oscillators, the parametric oscillator, and the frequency converter. The Wigner and tomographic representations of quantum states have also been important for the study of quantum systems. They have been used to distinguish between classical or nonclassical behavior of quantum states [18].

The Wigner quasiprobability distribution for a density operator $\hat{\rho }$ [9] is defined as:

$$W\left(q,p\right)=\frac{1}{2\pi }\int d\xi \;e^{-ip\xi }\langle q+\frac{\xi }{2}|\hat{\rho }|q-\frac{\xi }{2}\rangle$$

in a unit system, where ℏ→1. This function can be negative for some values of the variables *q* and *p*, where the negative values of W(q,p) depict a nonclassical behavior. The other important characteristic of the Wigner function is that its marginal functions are equal to the probability distributions for the position and momentum, i.e., P(q)=∫dpW(q,p) and P(p)=∫dqW(q,p).

The tomographic representation of a quantum state W(X) is defined as the Wigner quasiprobability distribution for the coordinate in a rotated and rescaled reference frame, where the new position operator reads $\hat{X}=s\cos\theta \;\hat{q}+s^{-1}\sin\theta \;\hat{p}$ and the new momentum is $\hat{P}=s^{-1}\cos\theta \;\hat{p}-s\sin\theta \;\hat{q}$, with s,θ∈R. The tomogram associated to the quantum state $\hat{\rho }$ is defined as:

(1) W(X)=∫dP∫W(q,p)δ(q−X)δ(P−p)dqdp.

Some properties of tomograms were considered in [19], while problems of employing tomography of Gaussian quantum states to discuss the system temperatures were mentioned in [20].

In the present work, we calculate some specific properties of thermal states (also known as Gibbs states). These states have a density operator written as $\hat{\rho }=\frac{e^{-\beta \hat{H}}}{\mathrm{Tr}(e^{-\beta \hat{H}})}$, with β=1/(kT), *k* being the Boltzmann constant and *T*, the temperature. This density matrix describes a particle or a system of particles, which are distinguishable from one another and the interaction between them is described by the Hamiltonian $\hat{H}$. The results presented in this work are also valid for one particle of a bigger *N*-particle system, in which the full statistic results can be obtained by the partition function of the whole system, which can be calculated as the product of the partition functions of the subsystems, i.e., $Z\left(T\right)=Z_{1}\left(T\right)\cdot Z_{2}\left(T\right)\cdots Z_{N}\left(T\right)$, with $Z_{j}\left(T\right)=\mathrm{Tr}\left(e^{-\beta \hat{H}}\right)$.

Our work is organized as follows, in Section 2, the Wigner and tomographic representations of a thermal state are studied in the one-dimensional quadratic case. The study of a temperature scale given by the properties of the state representation is also presented. Then, the Wigner and tomographic representations for a two-mode system thermal state are given in Section 3. The reduced Wigner functions and tomograms for each mode are then used to define a temperature scale for two harmonic oscillators coupled together. Finally, some concluding remarks are presented.

## 2. Wigner and Tomographic Representations of Unimodal Thermal States

The most general unimodal quadratic Hamiltonian can be written in the following form:

(2) $$\hat{H}=\frac{b}{2}\left({\hat{a}}^{†}\hat{a}+\frac{1}{2}\right)+\frac{c}{2}\left({\hat{a}}^{2}e^{i\phi }+{\hat{a}}^{†2}e^{-i\phi }\right)+d\hat{a}+d^{*}{\hat{a}}^{†}\;,$$

which eigenvectors $|\psi_{n}\rangle$ can be obtained by the following procedure. First, by using the translation $\hat{D}\left(\alpha \right)=\exp\left(\alpha {\hat{a}}^{†}-\alpha^{*}\hat{a}\right)$ and squeeze operator $S\left(\xi \right)=\exp(\frac{1}{2}\left(\xi^{*}{\hat{a}}^{†2}-\xi {\hat{a}}^{2}\right))$, it is possible to eliminate the linear part of the Hamiltonian of Equation (2) as:

$${\hat{H}}^{\prime }={\hat{D}}^{†}\left(\alpha \right)\hat{H}\hat{D}\left(\alpha \right)=\frac{b}{2}\left({\hat{a}}^{†}\hat{a}+\frac{1}{2}\right)+\frac{c}{2}\left({\hat{a}}^{2}e^{i\phi }+{\hat{a}}^{†2}e^{-i\phi }\right)+\kappa^{\prime },\;\alpha =\frac{4cde^{-i\phi }-2bd^{*}}{b^{2}-4c^{2}}\;,$$

where $\kappa^{\prime }$ is a constant, which does not contribute into the final result of our calculation. Then, we can obtain a diagonal Hamiltonian by using the squeeze operator; in other words,

(3) $$\begin{array}{ccc} & {\hat{H}}_{0}={\hat{S}}^{†}\left(\xi \right){\hat{H}}^{\prime }\hat{S}\left(\xi \right)=\frac{\gamma }{2}\left({\hat{a}}^{†}\hat{a}+\frac{1}{2}\right)+\kappa , & \\ & \gamma =\sqrt{b^{2}-4c^{2}},\;\xi =\frac{e^{-i\phi }}{4}\ln\left(\frac{b+2c}{b-2c}\right). \end{array}$$

As the eigenvectors of ${\hat{H}}_{0}$ are the standard Fock states |n〉, then the eigenvalues up to a constant of the original Hamiltonian $\hat{H}$ are given by $|\psi_{n}\rangle =\hat{D}\left(\alpha \right)\hat{S}\left(\xi \right)|n\rangle$, i.e.,

(4) $$\hat{H}|\psi_{n}\rangle =\frac{\gamma }{2}\left(n+\frac{1}{2}\right)|\psi_{n}\rangle .$$

This result leads us to the calculation of the thermal states at temperature *T* associated to the Hamiltonian $\hat{H}$,

(5) $$\hat{\rho }\left(\hat{H},T\right)=\frac{e^{-\hat{H}/T}}{\mathrm{Tr}(e^{-\hat{H}/T})}=\hat{D}\left(\alpha \right)\hat{S}\left(\xi \right)\frac{e^{-{\hat{H}}_{0}/T}}{\mathrm{Tr}(e^{-{\hat{H}}_{0}/T})}{\hat{S}}^{†}\left(\xi \right){\hat{D}}^{†}\left(\alpha \right),$$

and the density operators can be written as:

(6) $$\hat{\rho }\left(\hat{H},T\right)=\left(1-e^{-\frac{\gamma }{2T}}\right)\sum_{n=0}^{\infty }e^{-\frac{\gamma n}{2T}}|\psi_{n}\rangle \langle \psi_{n}|=\hat{D}\left(\alpha \right)\hat{S}\left(z\right)\left(1-e^{-\frac{\gamma }{2T}}\right)\sum_{n=0}^{\infty }e^{-\frac{\gamma n}{2T}}\left|n\rangle \langle n\right|{\hat{S}}^{†}\left(z\right){\hat{D}}^{†}\left(\alpha \right).$$

The properties of the state ${\hat{\rho }}_{0}=\left(1-e^{-\frac{\gamma }{2T}}\right)\sum_{n=0}^{\infty }e^{-\frac{\gamma n}{2T}}\left|n\rangle \langle n\right|$ can be used to obtain the properties of the state $\hat{\rho }$ by using the transformation $\hat{\rho }=\hat{D}\left(\alpha \right)\hat{S}\left(z\right){\hat{\rho }}_{0}{\hat{S}}^{†}\left(z\right){\hat{D}}^{†}\left(\alpha \right)$. For example, the covariance matrix and the mean values, which are the main properties of the Gaussian states, can be calculated in this way. It is straightforward that the covariance matrix (defined as $\sigma_{jk}=\frac{1}{2}\langle {\hat{o}}_{j}{\hat{o}}_{k}+{\hat{o}}_{k}{\hat{o}}_{j}\rangle -\langle {\hat{o}}_{j}\rangle \langle {\hat{o}}_{j}\rangle$ with $\hat{o}=\hat{p},\hat{q}$) and mean values can be written as:

(7) $$\sigma_{0}=\frac{1}{2}\coth\left(\frac{\gamma }{2T}\right)\left(\begin{array}{cc} \gamma & 0\\ 0 & \frac{1}{\gamma } \end{array}\right),\;{\langle \hat{x}\rangle }_{0}={\langle \hat{p}\rangle }_{0}=0.$$

The unitary transformation of the state $\hat{\rho }$ implies the symplectic transformation of the covariance matrix,

(8) $$\begin{array}{ccc} \; & \sigma =\frac{1}{2}\coth\left(\frac{\gamma }{2T}\right)\left(\begin{array}{cc} \gamma (\cosh(2r)-\sinh(2r)\cos\phi ) & \sinh(2r)\sin\phi \\ \sinh(2r)\sin\phi & \frac{1}{\gamma }\left(\cosh\left(2r\right)+\sinh\left(2r\right)\cos\phi \right) \end{array}\right), & \\ \; & \langle \hat{x}\rangle =-\frac{1}{\sqrt{2}}\left(\alpha +\alpha^{*}\right),\;\langle \hat{p}\rangle =\frac{i}{\sqrt{2}}\left(\alpha -\alpha^{*}\right) & \end{array}$$

with $r=\frac{1}{4}\ln\left(\frac{b+2c}{b-2c}\right)$ and $\alpha =\frac{4cde^{-i\phi }-2bd^{*}}{b^{2}-4c^{2}}$.

From the covariance matrix and mean values, the Wigner function and the symplectic or optical tomogram can be obtained for the thermal state. The Wigner function is written as:

$$W\left(x,p\right)=\frac{1}{2\pi \sqrt{\det\sigma }}\exp\left(-\frac{1}{2}\left(p-\langle \hat{p}\rangle ,x-\langle \hat{x}\rangle \right)\sigma^{-1}\left(\begin{array}{c} p-\langle \hat{p}\rangle \\ x-\langle \hat{x}\rangle \end{array}\right)\right),$$

and the area of the Wigner quasidistribution is given by the inverse of its second moment [21], i.e.,

(9) $$A_{W}=\frac{1}{{\;\int \int \;}_{-\infty }^{\infty }dp\;dx\;W^{2}\left(x,p\right)}=4\pi \sqrt{\det\sigma }.$$

The optical tomogram (symplectic tomogram with parameter s=1) can also be obtained using the covariance matrix; the result reads:

$$W\left(X,\theta \right)=\frac{1}{\sqrt{2\pi \sigma }}\exp\left[-\frac{{\left(X-\tilde{X}\right)}^{2}}{2\;\sigma }\right],$$

with $\sigma =\cos^{2}\theta \;\sigma_{qq}+\sin^{2}\theta \;\sigma_{pp}+\sin\left(2\theta \right)\sigma_{pq}$, and $\tilde{X}=\cos\theta \langle \hat{q}\rangle +\sin\theta \langle \hat{p}\rangle$.

The values for the area of the Wigner function and the width of the tomogram can be directly obtained. The area of the Wigner function of Equation (9) for the general covariance matrix given by Equation (8) can be calculated, resulting in the following expression:

(10) $$A_{W}\left(T\right)=2\pi \coth\left(\frac{\gamma }{2T}\right).$$

In our case, the values of parameters $\tilde{X}$ and σ of the thermal tomogram are functions of the temperature *T*, the phase θ, the parameter α, and the squeezing parameters *r* and ϕ, i.e.,

(11) $$\begin{array}{c} \sigma \left(T\right)=\frac{1}{2\gamma }\coth\left(\frac{\gamma }{2T}\right)(\sinh\left(2r\right)\left(\cos\phi \left(\cos^{2}\theta -\gamma^{2}\sin^{2}\theta \right)+\gamma \sin\left(2\theta \right)\sin\phi \right)+\\ \cosh\left(2r\right)\left(\gamma^{2}\sin^{2}\theta +\cos^{2}\theta \right)),\;\tilde{X}=\frac{1}{\sqrt{2}}\left(e^{i\theta }\alpha^{*}+e^{-i\theta }\alpha \right). \end{array}$$

When the temperature tends to zero, the thermal state depicts the ground state for the Hamiltonian $\hat{H}$, which is a pure state rather than a mixed thermal state. Intuitively, as the temperature rises the quantum state is formed by a mixture of more and more eigenstates of $\hat{H}$. For that reason, one can think that the area, which the thermal state has in the phase space, grows with the temperature. Because of that, one can suggest the area of the Wigner function as a way to measure the relative temperature of the system or, in other words, with this area is possible to define a temperature scale of a quantum system. The same idea can be applied to the simplectic or optical tomogram, the only difference is that the object measuring the relative temperature is the covariance of the tomogram σ. This hypothesis can be verified in the limit T≫1 as, in that limit, coth(γ/(2T))→2T/γ and, thus, the area of the Wigner function, Equation (10), and the width of the tomogram, Equation (11), are linear with the temperature in that limit (T≫1),

(12) $$\begin{array}{c} A_{W}\left(T\right)\to \frac{4\pi T}{\gamma },\;\;\sigma \left(T\right)\to \frac{T}{4\gamma^{2}}(\sinh\left(2r\right)\left(\cos\phi \left(\cos^{2}\theta -\gamma^{2}\sin^{2}\theta \right)+\gamma \sin\left(2\theta \right)\sin\phi \right)+\\ \cosh\left(2r\right)\left(\gamma^{2}\sin^{2}\theta +\cos^{2}\theta \right))\sinh\left(2r\right)). \end{array}$$

When *T* is small, we have a nonlinear behavior of $A_{W}\left(T\right)$ and σ(T) with the temperature. Nevertheless, they are still monotonic functions of the temperature.

**Figure 1:**
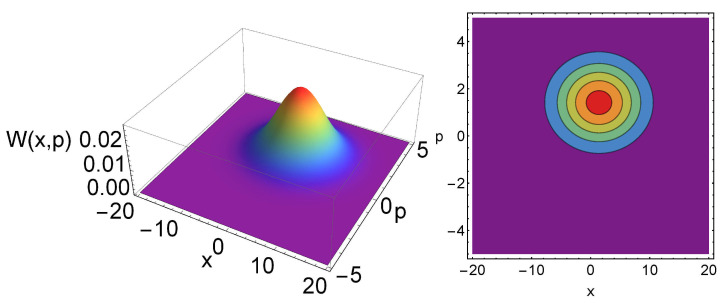
Wigner function (**left**) and its contour plot (**right**) for a thermal state with the parameters r=1, ϕ=0, T=10, $\gamma =\sqrt{3}$, α=1+i.

**Figure 2:**
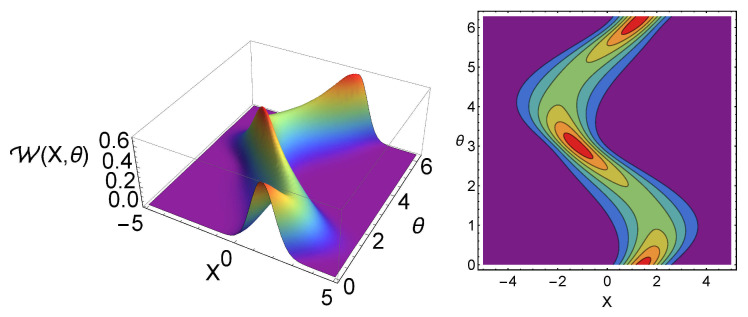
Optical tomogram W(X,θ) (**left**) and its contour for the state with parameters r=1/10, ϕ=π/3, γ=2, α=1+i, and temperature T=1 (**right**).

**Figure 3:**
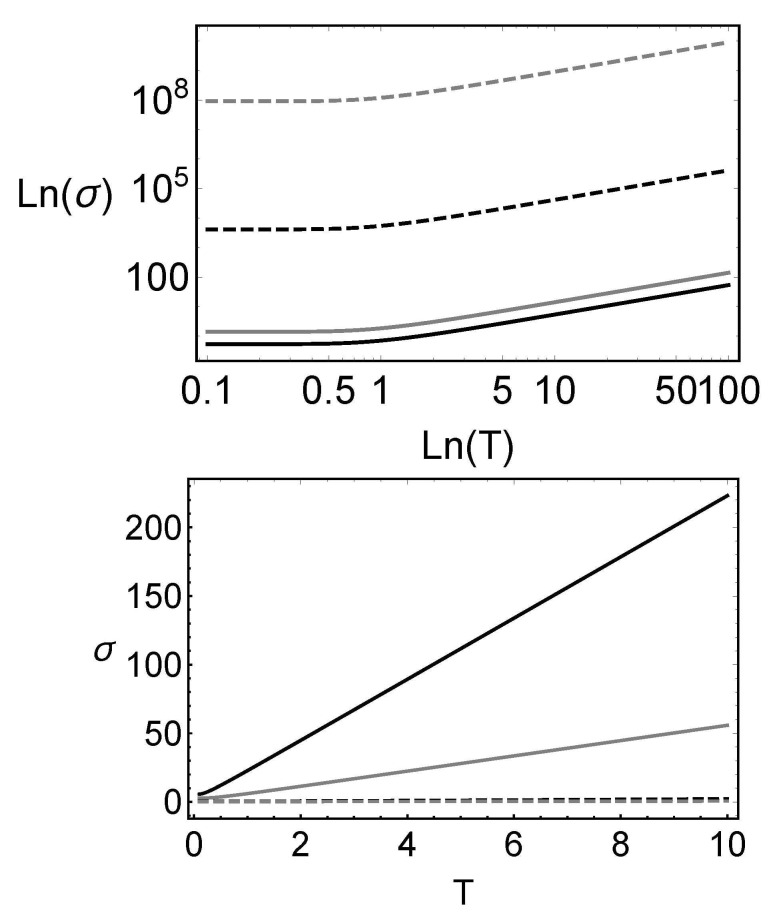
(**Top**) Logarithm of the covariance σ as a function of the logarithm of the temperature *T* for different values of the squeeze parameter r=0.5,1,5,10 shown in black, gray, dashed black, and dashed gray, respectively (γ=2, θ=0, ϕ=π/3) and (**bottom**) σ vs. *T* for different values of the energy parameter γ=0.5,1,5,10 shown in black, gray, dashed black, and dashed gray, respectively (r=1, θ=0, ϕ=π/3).

The dependences of the Wigner function and the tomogram in terms of the temperature are shown in Figure 1 and Figure 2, respectively. Here, one can notice the Gaussian nature of the Wigner function and the oscillating behavior of the tomogram. One can also show that the width of the tomogram in the *X* direction is proportional to the temperature of the system. In other words, one can measure the temperature of the system by measuring the tomogram width. In Figure 3, the plots of the tomogram as functions of the temperature for different squeezing parameters *r* and different energy parameter γ, are presented. In all the plots, the linear temperature dependence of σ can be inferred for T>1, making the measurement of this quantity be a possible experimental way to obtain the temperature of quantum states.

## 3. Tomographic Representation of Two-Mode Thermal States

In this section, we present general aspects of how to calculate the covariance matrix of a general two-dimensional Gibbs thermal state, whose interaction is given by a quadratic Hamiltonian. For this, we establish a series of transformations which allow us to write the two-mode Hamiltonian in a simplified form. After that, the covariance matrix of the corresponding thermal state is calculated and then the Wigner and tomographic representations of the state are calculated.

The general two-mode Hamiltonian considered in the study is the following:

(13) $$\hat{H}=\left({\hat{p}}_{1},{\hat{q}}_{1},{\hat{p}}_{2},{\hat{q}}_{2}\right)\left(\begin{array}{cccc} \omega_{11} & \omega_{12} & \omega_{13} & \omega_{14}\\ \omega_{12} & \omega_{22} & \omega_{23} & \omega_{24}\\ \omega_{13} & \omega_{23} & \omega_{33} & \omega_{34}\\ \omega_{14} & \omega_{24} & \omega_{34} & \omega_{44} \end{array}\right)\left(\begin{array}{c} {\hat{p}}_{1}\\ {\hat{q}}_{1}\\ {\hat{p}}_{2}\\ {\hat{q}}_{2} \end{array}\right),$$

where all the coefficients are real. By using the series of the local symplectic transformations as rotations $\hat{R}\left(\theta \right)=e^{-i\theta_{i}{\hat{a}}_{i}^{†}{\hat{a}}_{i}}$ and squeezing $\hat{D}\left(\xi_{i}\right)=e^{\frac{1}{2}\left(\xi_{i}^{*}{\hat{a}}_{i}^{2}-\xi_{i}{\hat{a}}_{i}^{†2}\right)}$ (i=1,2), it is possible to take the general Hamiltonian of Equation (13) into a simplified form,

(14) $${\hat{H}}^{\prime }=\mu_{11}\left({\hat{P}}_{1}^{2}+{\hat{Q}}_{1}^{2}\right)+\mu_{22}\left({\hat{P}}_{2}^{2}+{\hat{Q}}_{2}^{2}\right)+\mu_{13}{\hat{P}}_{1}{\hat{P}}_{2}+\mu_{24}{\hat{Q}}_{1}{\hat{Q}}_{2}.$$

From this transformed Hamiltonian, one can use the expression for the propagator of the Hamiltonian $G\left(x^{\prime },x,t\right)=\langle x^{\prime }|e^{-i\hat{H}t}|x\rangle$, given in [22,23,24] and, after the change of the time by the temperature it→1/T, one can see that the exponential of the Hamiltonian $\hat{H}$: $e^{-\hat{H}/T}$ can be written in the position representation as follows:

(15) $$\langle x^{\prime }|e^{-\hat{H}/T}|x\rangle =e^{-\tilde{x}a_{1}x+\tilde{x}a_{2}x^{\prime }-{\tilde{x}}^{\prime }a_{3}x^{\prime }},$$

where $a_{1,2,3}$ are 2×2 matrices. From these expressions, it is possible to retrieve the covariance matrix of the system. As reported in previous work [25,26], the covariance matrix elements, in the position representation, are written in terms of the parameters of a general two-mode density matrix. By using these results, the covariance matrix is given in the standard expression [27]:

(16) $$\sigma \left(T\right)=\left(\begin{array}{cc} S_{1} & 0\\ 0 & S_{2} \end{array}\right)\left(\begin{array}{cccc} a_{1} & 0 & b_{1} & 0\\ 0 & a_{1} & 0 & b_{2}\\ b_{1} & 0 & a_{2} & 0\\ 0 & b_{2} & 0 & a_{2} \end{array}\right)\left(\begin{array}{cc} {\tilde{S}}_{1} & 0\\ 0 & {\tilde{S}}_{2} \end{array}\right),$$

where $S_{1,2}$ are symplectic local operations associated to the local rotations and squeezing transformations described above. Specifically the symplectic operations have the form:

(17) $$S_{i}=\left(\begin{array}{cc} \cos\theta_{i} & \sin\theta_{i}\\ -\sin\theta_{i} & \cos\theta_{i} \end{array}\right)\;\mathrm{or}\;S_{i}=\left(\begin{array}{cc} e^{-z_{i}} & 0\\ 0 & e^{z_{i}} \end{array}\right),$$

for rotations or squeezing, respectively. We point out that the temperature dependence of the results is in the set of parameters ${\theta_{i},a,b,z}_{i}$ (i=1,2). The Wigner and tomographic representations of the thermal Gibbs states can then be calculated. It is known that [28], for any Gaussian state, the Wigner functions are written as:

(18) $$\begin{array}{c} W\left(x_{1},p_{1},x_{2},p_{2}\right)=\frac{1}{4\pi^{2}\sqrt{\det\sigma }}\exp\left(-\frac{1}{2}\left(p_{1},x_{1},p_{2},x_{2}\right)\sigma^{-1}\left(\begin{array}{c} p_{1}\\ x_{1}\\ p_{2}\\ x_{2} \end{array}\right)\right). \end{array}$$

The reduced Wigner functions for each one of the modes can be obtained, by using the covariance matrix of each subsystem,

$$\sigma_{i}=\left(\begin{array}{cc} \sigma_{p_{i}p_{i}} & \sigma_{p_{i}q_{i}}\\ \sigma_{p_{i}q_{i}} & \sigma_{q_{i}q_{i}} \end{array}\right),\;W_{i}\left(x,p\right)=\frac{1}{2\pi \sqrt{\det\sigma_{i}}}\exp\left(-\frac{1}{2}\left(x,p\right)\sigma_{i}^{-1}\left(\begin{array}{c} x\\ p \end{array}\right)\right),\;i=1,2.$$

The Wigner function area for each one of the modes can be written as Equation (9) which, in this case, reads:

(19) $$A_{W_{i}}=\frac{1}{{\;\int \int \;}_{-\infty }^{\infty }dxdp\;W_{i}^{2}\left(x,p\right)}=4\pi \sqrt{\det\sigma_{i}}$$

From the definition of the tomogram in terms of the Wigner function, it is possible to obtain the tomographic representation of the Gibbs states as:

(20) $$W_{i}\left(X,\phi \right)=\frac{1}{\sqrt{2\pi S_{i}}}\exp\left(-\frac{X^{2}}{2S_{i}}\right),\;S_{i}=s^{2}\cos^{2}\phi \;\sigma_{q_{i}q_{i}}+s^{-2}\sin^{2}\phi \;\sigma_{p_{i}p_{i}}+\sin\left(2\phi \right)\;\sigma_{p_{i}q_{i}},$$

where *s* and ϕ are the tomogram parameters.

Then, we can obtain the definition of the temperature scale, using several properties listed above; for example, using the two-mode Wigner function and its area, or using the two-mode tomogram, the reduced Wigner functions, or the reduced tomograms. To exemplify this richness, we provide further calculations for a specific Hamiltonian of two coupled harmonic oscillators.

### Example

Recently, the Hamiltonian for two coupled harmonic oscillators has been relevant given the applications in several problems. For example, in [29,30], the general time-dependent solutions for this Hamiltonian were found. In [31], the entanglement between modes in this particular system was reported, while in [32], the reflection coefficient in such a type of system was presented.

#### Two Coupled Harmonic Oscillators

Here, we present two coupled harmonic oscillators with Hamiltonians given by the following expression:

(21) $$\hat{H}=\frac{1}{2}\left({\hat{p}}_{1}^{2}+\omega_{1}^{2}q_{1}^{2}\right)+\frac{1}{2}\left({\hat{p}}_{2}^{2}+\omega_{2}^{2}q_{2}^{2}\right)-C{\hat{q}}_{1}{\hat{q}}_{2},$$

with *C* being the coupling parameter. This particular Hamiltonian can be expressed as a sum of two harmonic oscillators by employing the transformation $\hat{U}=e^{-i\alpha ({\hat{q}}_{1}{\hat{p}}_{2}-{\hat{q}}_{2}{\hat{p}}_{1})}$ [33], with $\tan\left(2\alpha \right)=C/\left(\omega_{1}^{2}-\omega_{2}^{2}\right)$. In other words, the Hamiltonian after the transformation can be written as:

(22) $${\hat{H}}^{\prime }=\frac{1}{2}\left(p_{1}^{\prime 2}+\Omega_{1}^{2}q_{1}^{\prime 2}+p_{2}^{\prime 2}+\Omega_{2}^{2}q_{2}^{\prime 2}\right),$$

with $\Omega_{1}=\omega_{1}^{2}\cos^{2}\alpha +\omega_{2}^{2}\sin^{2}\alpha +\frac{C}{2}\sin\left(2\alpha \right)$ and $\Omega_{2}=\omega_{2}^{2}\cos^{2}\alpha +\omega_{1}^{2}\sin^{2}\alpha -\frac{C}{2}\sin\left(2\alpha \right)$. Then the resulting covariance matrix of the system is:

(23) $$\sigma =\frac{1}{2}\left(\begin{array}{cccc} a_{1} & 0 & c_{1} & 0\\ 0 & a_{2} & 0 & c_{2}\\ c_{1} & 0 & b_{1} & 0\\ 0 & c_{2} & 0 & b_{2} \end{array}\right),$$

with:

(24) $$\begin{array}{c} a_{1}=\Omega_{1}\cos^{2}\alpha \coth\left(\frac{\Omega_{1}}{2T}\right)+\Omega_{2}\sin^{2}\alpha \coth\left(\frac{\Omega_{2}}{2T}\right),\;a_{2}=\frac{\cos^{2}\alpha \coth\left(\frac{\Omega_{1}}{2T}\right)}{\Omega_{1}}+\frac{\sin^{2}\alpha \coth\left(\frac{\Omega_{2}}{2T}\right)}{\Omega_{2}},\\ b_{1}=\Omega_{2}\cos^{2}\alpha \coth\left(\frac{\Omega_{2}}{2T}\right)+\Omega_{1}\sin^{2}\alpha \coth\left(\frac{\Omega_{1}}{2T}\right),\;b_{2}=\frac{\cos^{2}\alpha \coth\left(\frac{\Omega_{2}}{2T}\right)}{\Omega_{2}}+\frac{\sin^{2}\alpha \coth\left(\frac{\Omega_{1}}{2T}\right)}{\Omega_{1}},\\ c_{1}=\frac{1}{2}\sin\left(2\alpha \right)\left(\Omega_{2}\coth\left(\frac{\Omega_{2}}{2T}\right)-\Omega_{1}\coth\left(\frac{\Omega_{1}}{2T}\right)\right),\;c_{2}=\frac{\sin\left(2\alpha \right)\left(\Omega_{1}\coth\left(\frac{\Omega_{2}}{2T}\right)-\Omega_{2}\coth\left(\frac{\Omega_{1}}{2T}\right)\right)}{2\Omega_{1}\Omega_{2}}. \end{array}$$

The Wigner and tomographic representations of the thermal states can be calculated and their properties obtained. For example, the area of the total Wigner function reads:

(25) $$A_{W}\left(T\right)=4\pi^{2}\coth\left(\frac{\Omega_{1}}{2T}\right)\coth\left(\frac{\Omega_{2}}{2T}\right);$$

we can see that this expression cannot allow us to have a linear temperature scale, as even for T≫1, this expression is quadratic in the temperature $A_{W}\left(T\right)\to 16\pi^{2}T^{2}/\left(\Omega_{1}\Omega_{2}\right)$. This behavior is expected as the area for the two-mode Wigner function measures the size of the distribution in a four-dimensional space rather than the two-dimensional space measured by the reduced Wigner areas. The areas of the reduced Wigner functions are:

(26) $$\begin{array}{c} A_{W_{1}}\left(T\right)=\frac{2\pi }{\sec^{2}\alpha }\sqrt{\frac{\left(\Omega_{2}\coth\left(\frac{\Omega_{1}}{2T}\right)+\Omega_{1}\coth\left(\frac{\Omega_{2}}{2T}\right)\tan^{2}\alpha \right)\left(\Omega_{1}\coth\left(\frac{\Omega_{1}}{2T}\right)+\Omega_{2}\coth\left(\frac{\Omega_{2}}{2T}\right)\tan^{2}\alpha \right)}{\Omega_{1}\Omega_{2}}},\\ A_{W_{2}}\left(T\right)=\frac{2\pi }{\sec^{2}\alpha }\sqrt{\frac{\left(\Omega_{2}\coth\left(\frac{\Omega_{2}}{2T}\right)+\Omega_{1}\coth\left(\frac{\Omega_{1}}{2T}\right)\tan^{2}\alpha \right)\left(\Omega_{1}\coth\left(\frac{\Omega_{2}}{2T}\right)+\Omega_{2}\coth\left(\frac{\Omega_{1}}{2T}\right)\tan^{2}\alpha \right)}{\Omega_{1}\Omega_{2}}}. \end{array}$$

**Figure 4:**
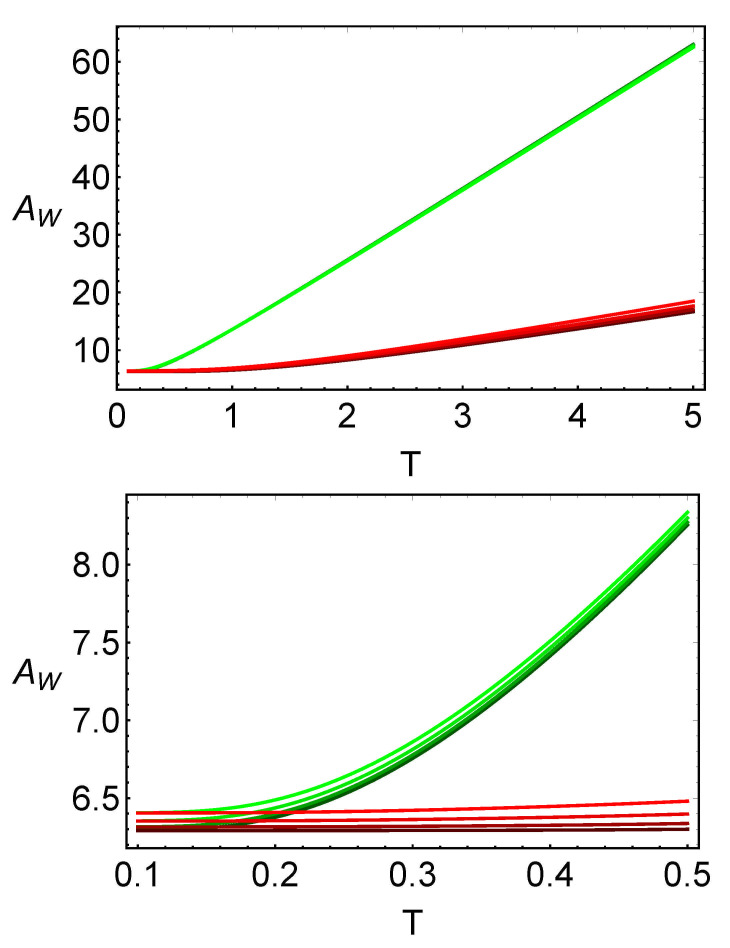
Area for the reduced Wigner quasiprobability distribution as functions of the temperature (**top**) and a zoom (**bottom**). The different shades of red (green) denote the first (second) subsystem for C=1/10,2/10,3/10,4/10. The darkest color for each mode denotes a coupling constant C=1/10 and the lightest one, a coupling constant C=4/10. In all cases, the frequencies are $\omega_{1}=1$ and $\omega_{2}=2$.

From these expressions, one can conclude that the area of the reduced Wigner function is a good candidate to measure the temperature of a thermal quantum system. As discussed in the previous section, these areas linearly vary with the temperature in the case T≫1. The area of the reduced Wigner functions is plotted in Figure 4, where one can see that the areas of both reduced Wigner functions are always proportional to the temperature and, thus, this area can be used to detect relative temperatures of a system given at two fixed points. It is also seen in the figure that the differences between the plots for different values of the parameter *C* are closely related.

In principle, any of these reduced Wigner areas can be chosen in order to define a temperature scale as they have a similar behavior for T>1. In Figure 4, one can see that the numerical results for both the areas will differ between the two modes. In the case where the two areas differ greatly from one another, one can choose between them by taking into account which subsystem area grows slower with the temperature; as in an experiment, one would reconstruct the reduced Wigner function with less measurements. Then, a linear temperature scale can be defined by using two reference points (as we need two points to define a line). For example, we can measure the area at 0 ${}^{\circ }$C and at 100 ${}^{\circ }$C, and from them, we can infer the temperature of the system for any other value of the area between the two points.

The tomographic representation of both reduced states is given by Equation (20) with covariances:

(27) $$\begin{array}{c} S_{1}=\frac{\cos^{2}\alpha \coth\left(\frac{\Omega_{1}}{2T}\right)\left(\Omega_{1}^{2}\sin^{2}\theta +\cos^{2}\theta \right)}{2\Omega_{1}}+\frac{\sin^{2}\alpha \coth\left(\frac{\Omega_{2}}{2T}\right)\left(\Omega_{2}^{2}\sin^{2}\theta +\cos^{2}\theta \right)}{2\Omega_{2}},\\ S_{2}=\frac{\cos^{2}\alpha \coth\left(\frac{\Omega_{2}}{2T}\right)\left(\Omega_{2}^{2}\sin^{2}\theta +\cos^{2}\theta \right)}{2\Omega_{2}}+\frac{\sin^{2}\alpha \coth\left(\frac{\Omega_{1}}{2T}\right)\left(\Omega_{1}^{2}\sin^{2}\theta +\cos^{2}\theta \right)}{2\Omega_{1}}. \end{array}$$

**Figure 5:**
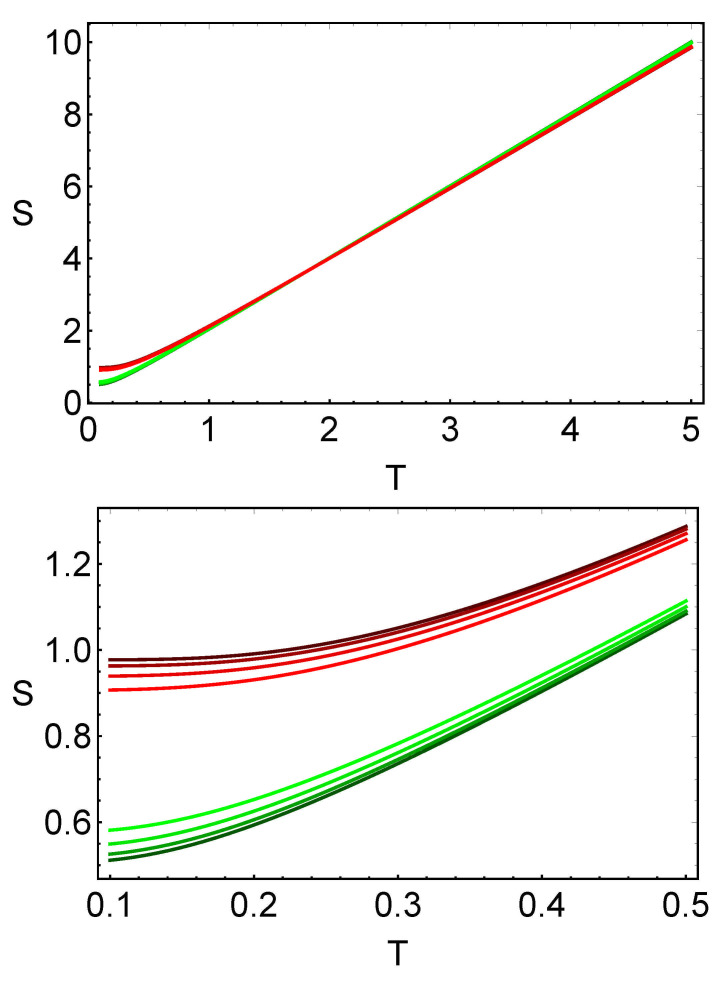
Covariance for the reduced tomographic representation as a function of the temperature (**top**) and a zoom (**bottom**). The different shades of red (green) denote the first (second) subsystem for C=1/10,2/10,3/10,4/10. The darkest color for each mode denotes a coupling constant C=1/10 and the lightest, a coupling constant C=4/10. In all the cases, the frequencies $\omega_{1}=1$ and $\omega_{2}=\sqrt{2}$, and the angle $\theta =\sqrt{2}$ were used.

As in the case of the Wigner function, the tomographic representation can be used to define a temperature scale by using the covariance (the tomogram width). In Figure 5, one can see that there is a zone, where the covariance for each mode is almost a linear function of the temperature, in which a temperature scale can be defined. As in the case of the Wigner function, the widths of the tomograms do not strongly depend on the parameter *C* for the specific examples shown in the figure.

## 4. Concluding Remarks

To summarize, we point out the main results of our work.

We presented a small review of the probability representation of quantum states, where the particle states are described by fair tomographic-probability distribution functions. Concrete examples of thermal-equilibrium states of one-mode and two-mode oscillators at temperature *T* were studied, and explicit expressions for the state symplectic tomograms were calculated. These tomograms are given by Gaussian conditional probability distribution functions. Tomograms of the classical oscillator states describing their thermal-equilibrium states are also given by Gaussian functions.

By considering one-dimensional and two-dimensional quadratic systems, the expressions for the covariance matrix, the Wigner function, and the symplectic tomogram were discussed. The area of the Wigner function and the width of the symplectic tomogram were proposed as possible measures of the temperature of the mentioned systems and this hypothesis was confirmed for the general one-dimensional case and for a particular two-dimensional system of coupled harmonic oscillators. It was found that, for T≫1, the area of the one-mode thermal Gaussian systems, the area of the Wigner function, and the covariance of the tomogram follow a linear dependence on the temperature. It was also found that for T≪1, the dependence is nonlinear, but the resulting function shows a monotonic grown with the temperature. We point out that these results can be used for the experimental measurement of the temperature by using the Wigner and tomographic representations of thermal states.

## Data Availability

Not applicable.
